# QoS Aware and Fault Tolerance Based Software-Defined Vehicular Networks Using Cloud-Fog Computing

**DOI:** 10.3390/s22010401

**Published:** 2022-01-05

**Authors:** Sidra Abid Syed, Munaf Rashid, Samreen Hussain, Fahad Azim, Hira Zahid, Asif Umer, Abdul Waheed, Mahdi Zareei, Cesar Vargas-Rosales

**Affiliations:** 1 Department of Biomedical Engineering, Faculty of ESTM, Ziauddin University, Karachi 74600, Pakistan; sidra.agha@yahoo.com (S.A.S.); hira.zahid@zu.edu.pk (H.Z.); 2 Department of Electrical and Software Engineering, Faculty of ESTM, Ziauddin University, Karachi 74600, Pakistan; softdepartment@yahoo.com; 3 Begum Nusrat Bhutto Women University, Sukkur 65400, Pakistan; solution.sam@yahoo.com; 4 Department of Electrical Engineering, Faculty of ESTM, Ziauddin University, Karachi 74600, Pakistan; engr_dean@yahoo.com; 5 Department of Computer Science & Information Technology, Hazara University, Mansehra 21120, Pakistan; asifumer@hu.edu.pk; 6 Department of Computer Science, Northern University, Nowshera 24100, Pakistan; 7 School of Electrical and Computer Engineering, Seoul National University, Seoul 08826, Korea; 8 Tecnologico de Monterrey, School of Engineering and Sciences, Zapopan 45201, Mexico; m.zareei@tec.mx (M.Z.); cvargas@tec.mx (C.V.-R.)

**Keywords:** vehicular ad-hoc network, quality of service, priority basis scheduling, safety/non-safety messages, response time, fault-tolerance

## Abstract

Software-defined network (SDN) and vehicular ad-hoc network (VANET) combined provided a software-defined vehicular network (SDVN). To increase the quality of service (QoS) of vehicle communication and to make the overall process efficient, researchers are working on VANET communication systems. Current research work has made many strides, but due to the following limitations, it needs further investigation and research: Cloud computing is used for messages/tasks execution instead of fog computing, which increases response time. Furthermore, a fault tolerance mechanism is used to reduce the tasks/messages failure ratio. We proposed QoS aware and fault tolerance-based software-defined V vehicular networks using Cloud-fog computing (QAFT-SDVN) to address the above issues. We provided heuristic algorithms to solve the above limitations. The proposed model gets vehicle messages through SDN nodes which are placed on fog nodes. SDN controllers receive messages from nearby SDN units and prioritize the messages in two different ways. One is the message nature way, while the other one is deadline and size way of messages prioritization. SDN controller categorized in safety and non-safety messages and forward to the destination. After sending messages to their destination, we check their acknowledgment; if the destination receives the messages, then no action is taken; otherwise, we use a fault tolerance mechanism. We send the messages again. The proposed model is implemented in CloudSIm and iFogSim, and compared with the latest models. The results show that our proposed model decreased response time by 50% of the safety and non-safety messages by using fog nodes for the SDN controller. Furthermore, we reduced the execution time of the safety and non-safety messages by up to 4%. Similarly, compared with the latest model, we reduced the task failure ratio by 20%, 15%, 23.3%, and 22.5%.

## 1. Introduction

The recent development of information communication technology (ICT), vehicular networks (VN), and their communication systems has attracted researchers’ attentions. The advanced communication system of vehicles is especially used for efficient road traffic and safety. The vehicular ad-hoc network (VANET) mainly consists of three communication types as vehicle to vehicle (V2V), vehicle to road (V2R), and vehicle to infrastructure (V2I) [1]. The communication techniques that are used by VANET are the dedicated short-range communication (DSRC) method and wireless access in vehicular environments (WAVE). Current research work is in progress on designing or developing more efficient communication techniques. However, VANET communication faces several difficulties: scalability, flexibility, security, and less programmability [2]. To overcome the above challenges, researchers developed a new paradigm known as software-defined networking (SDN). The said technology is used to separate control plan from data plan and provide programmability to the communication system of VANET. Due to SDN, the dynamic communication of messages is activated, and the overall system becomes more attractive as required by the public [3]. The above technology provides a decent Application programming interface (APIs) and provides new facilities and business interpretations. SDN is unique, flexible, programmable, and centralized dynamic control of communication. Due to the several pros of SDN, researchers combined SDN and VANET for better communication among vehicles, which is known as a software defined vehicular network (SDVN) [4,5]. To make the communications systems of vehicles on the road, SDVN plays a vital role. Most of the time, when there is less traffic and movement on roads, then there is also the problem of unwanted acts like theft, security, etc.; so, in this case, the message of the vehicle must reach the monitoring cells (i.e., the police) [6]. If, in such cases, vehicle messages fail, then the tourists/passengers may be in danger.

The data generated by SDVN are stored on the cloud for further processing and storage. Cloud computing is defined as “a model for enabling ubiquitous, convenient, on-demand network access to a shared pool of configurable computing resources (e.g., networks, servers, storage, applications, and services) that can be rapidly provisioned and released with minimal management effort or service provider interaction. This cloud model comprises five essential characteristics, three service models, and four deployment models” [7]. We need to use fog computing to reduce response time to store and execute SDVN vehicles’ data. Fog computing minimizes delay, and the servers are placed to nearby edges for the users. Fog computing is defined as “a geographically distributed computing architecture with a resource pool consists of one or more ubiquitously connected heterogeneous devices (including edge devices) at the edge of the network and not exclusively seamlessly backed by cloud services, to collaboratively provide elastic computation, storage and communication (and many other new services and tasks) in isolated environments to a large scale of clients in proximity” [8]. Current models of SDVN performed well, but they are using the cloud for execution and storage, which will increase the response time of critical/priority-based tasks. Furthermore, the main challenge is fault tolerance, as if priority/critical-based tasks fail but the sender is not aware of it, it will create problems for other vehicles in the attached area.

### Contributions

1. A new architecture is proposed based on SDVN; fog nodes are used instead of cloud computing to reduce response time.
2. Response time is considered in the proposed model, which is an important QoS parameter of the vehicles messages/tasks/data.
3. Fault-tolerance-based architecture is provided to reduce tasks/messages failure ratio.
4. Message priority is calculated based on the nature of messages instead of deadline and size, as several highest/critical messages have more size, but its nature remains critical/urgent. 

Furthermore, the paper is outlined as follows: Section 2 presents literature review. Section 3 consists of the problem formulation; Section 4 consists of the proposed solution. Section 5 consists of simulation setup and results. Section 6 is discussed, while Section 7 is a conclusion of the paper.

## 2. Literature Review

In this section, detailed literature is provided about the proposed model. The literature is divided into VANET models, SDN-based models, SDVN-based models, and scheduling SDVN models, as shown in Table 1.

Table 1 models are further explained in detail in the following subpart of the paper:

### 2.1. VANET Models

An intelligent transportation system (ITS) is an advanced communication system amongst vehicles on the roadside to exchange information. Its advanced type is VANET, which can connect thousands of wireless nodes (Vehicles). VANET is an advanced type of ad-hoc network, which is used by ITS currently. In [3], the author proposed the VANET architecture-based mobile ad-hoc network (MANET). The proposed model is used for vehicles by using wireless technology to decrease energy consumption while using dynamic topology. Similarly, in [9], the author proposed safety and non-safety-based VANET architecture. In the proposed model, the author sent safety messages first and then non-safety messages to increase QoS.  In VANET, vehicles are directly connected to share necessary information.

### 2.2. SDN-Based Models

According to [2], SDN is more efficient than traditional networks, as the SDN data plane is separate from the control plane. In SDN, the centralized controller can view the complete network and manage network traffic efficiently compared to traditional networks. Similarly, in [10], the author proposed the Roadside Unit (RSU)-based SDN model, in which they used CR technology to transmit the vehicle information by using the 5G networking model. In [11], the author proposed the Software-Defined Network Edge (SDNE) model. The proposed model provided network services to the edge of networking devices to reduce communication delay and respond quickly. They provided a three-tier architecture that performs very well in terms of response time and energy consumption. 

### 2.3. SDVN-Based Models

SDVN is based on SDN and VANET architectures. SDVN is not fully centralized, as it is partially centralized and has hierarchical architecture. In [5], the author proposed a topology-based routing protocol for SDVN. The proposed model is used for vehicle dynamic path selection for communication among other vehicles in real time. They used predicated topologies to maintain routing tables and paths. They used two communications models such as uni-cast for communication and geo-cast for data dissemination. The proposed SDVN model performed well in terms of communication and dissemination of information. Similarly, in [12], the authors proposed and placed dynamic controllers in the edges of SDVN architecture. The proposed model performed well in road traffic. The proposed model reduced network changes as compared with the available network models. In [13], the authors proposed multi-access edge computing (MEC) for vehicles. They used two communication models: information/messages receiving, while the other is for messages forwarding to the connected vehicles. Model one is used for information forwarding to V2V and V2I. The second model is used for updating information and forwarding information using the Open-Flow protocol. The proposed model consists of four layers and reduces the latency of communication messages, and improves the routing path. In [14], the author proposed a prototype-based SDVN architecture in which the author examined backbone real hardware that consists of Open-Flow switches. In [15], the proposed RTISAR model reduces delay and packet loss, and makes overall communication from source to destination. In [16], the author proposed an application layer for VANET vehicles to reduce communication delay and manage massive traffic in ruler and urban areas. 

### 2.4. Scheduling SDVN Models

Scheduling is the process of allocating tasks/messages for communication to take place in order. As the local SDN control receives multiple messages, such scheduling mechanisms must manage and send messages based on priority. In some cases, we need to send essential data earlier than the already received messages for transmission, so in this situation, we need such scheduling that gives proper priority to messages and then transmits it based on the attached priorities. In [17], the author proposed the Unmanned Aerial Vehicle (UAV) model. The proposed model is infrastructure-less and based on SDN technology. The author provided a theoretical offloading mechanism in which they offload data based on different levels of zones. Emergency zone data are sent first, then the other zone data, and so on. The main aspect of the proposed model is to keep a balance between energy consumption and communication delay. Similarly, in [18], the author proposed a priority-based scheduling algorithm called the RSA algorithm tapping vigorous cloud. The proposed model divides the data into emergency data, and least used data, urgent data, and the average user data. They further send the data according to the scheduling algorithms such as FCFS, NDS, and SJF. The proposed model performed well in terms of energy consumption and bandwidth, as shown in the results. In [19], the authors proposed scheduling algorithm-based deadlines and size. If any task/message deadline and size is less than the model, send that data first, and so on. They provided D*S Algorithm by sending vehicle messages to RSU. The proposed model calculates message priority based on deadline and size. In [20], they proposed a collective scheduling algorithm in which they categorized messages into three categories: the size of messages, static factor, and dynamic factor. Static messages are divided into safety and no safety messages, while dynamic messages are calculated from VANET clustering. From the results of the provided mechanism, energy consumption is decreased and performed well. Zhu et al. [21] proposed the Hybrid Emergency Message Transmission (HEMT) model by using SDN technology. Similarly, ref. [22] proposed a cloud-based scheduling model. The proposed model is based on the nature of data such as video, audio, and text. In [23,24], the authors proposed a mobility-aware scheduling algorithm by sending and receiving vehicle messages in the concerned cluster circle. The authors of [25] proposed a Markov-based model while giving high priority to high mobility vehicles. Similarly, in [26], the author proposed a packet-based scheduling algorithm by using a multi-level queuing system. Another scheduling algorithm is provided in which the authors proposed a scheduling model and gave high priority to the packets whose deadline is near to expire. In [27,28], the authors recently proposed efficient architecture for SDVN by using a priority basis scheduling algorithm for time-critical and non-critical messages. Its main limitation is that they did not use fog instead of cloud to decrease response time, and they also did not use a fault tolerance mechanism to make the overall process of SDVN communication efficient.

Furthermore, in [29], the author proposed vehicle to vehicle, vehicle to infrastructure communication for congestion avoidance. They used linear adaptive congestion control designed for leveraging the performance of VANET communication. They did not use a fault tolerance mechanism for data offloading and fog for data processing to reduce delay as compared with the proposed model. Similarly, in [30] the author proposed QoS aware vehicle communication model using the clustering technique. They reduced packet drop ratio and messages delay. They did not use nearby edge devices for processing to reduce the further delay of the messages.

Based on current literature, we investigated and found the following limitations of SDVN communications:

- Response time of messages/data need to reduce
- Fault tolerance mechanism should be used to reduce tasks/messages failure ratio
- Mobility aware protocols need to develop for SDVN communications
- Cache-based scheduling algorithms needed to reduce energy consumption

## 3. Problem Formulation

**Figure 1:**
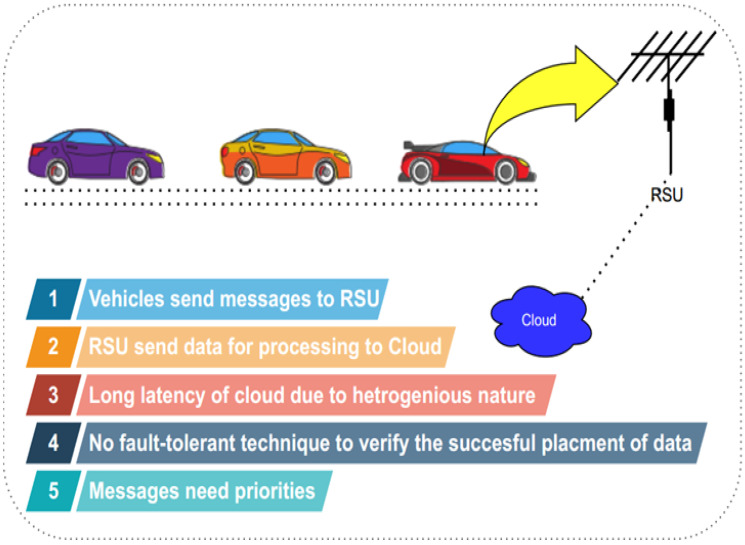
Problem Formulation-1.

As reflected from the Literature Review [5,9,10,11,12,13,14,15,16,17,18,19,20,21,22,23,24,25,26,27,28,31], they used the Cloud for data execution and storage, which has the following limitations, as shown in Figure 1:

1. Response time is not provided for the safety and non-safety messages of vehicles
2. Cloud is used for task execution, which leads to higher response time
3. Safety messages are given priority based on deadline and size, but not given priority based on message nature, as some messages are large in size, but have the highest priority.

Furthermore, it is observed that no fault-tolerance method is used in [27,28]; when any highest priority message does not deliver to the destination, then it may create an efficiency problem of the proposed model.

## 4. Proposed Solution

In this section, the proposed model is provided as follows:

### 4.1. System Architecture

**Figure 2:**
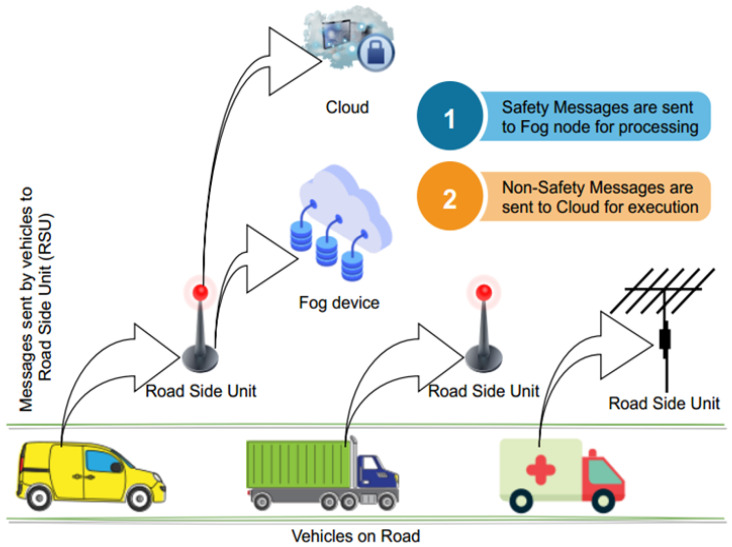
System Architecture of the proposed model.

The system architecture of the proposed model is provided here in detail as follows, and as shown in Figure 2:

### 4.2. SDN Based Smart Gateway

Software-defined network smart gateway is a gateway that receives vehicles’ messages/data and processes it according to rules embedded in it, and forwards it to other vehicles when required, and sends to the cloud or fog for storage and processing. Smart gateways are placed in fog nodes near the roadside to reduce response time.

### 4.3. VANET Vehicles

VANET vehicles are part of SDVN and communicate with each other during critical times, or share any important information.

#### 4.3.1. SDN Controller

The SDN controller is used for information/messages prioritization and forwarding to destination. The controller is connected to the nearby SDN nodes and, from time to time, updates routing tables of the received information. The controller is placed in edge fog nodes to get the processing power and short storage. For heavy computation and permanent storage, the controller sends information to the cloud.

#### 4.3.2. Fog Nodes

Fog nodes are used to provide nearby processing and storage to VANET Vehicles messages/information. SDN nodes and the main SDN controller are placed on fog nodes to reduce response time.

#### 4.3.3. Cloud Datacenter

The Cloud is used for heavy computation and permanent storage when required by VANET information/messages.

### 4.4. QAFT-SDVN Proposed Model

**Figure 3:**
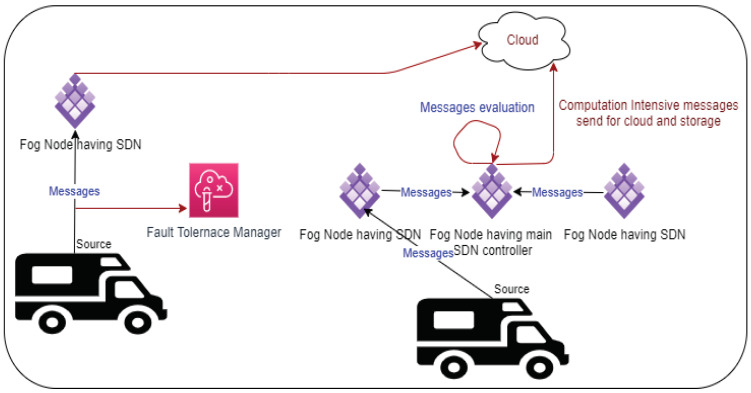
QAFT-SDVN Proposed Model.

The proposed model consists of vehicles that will communicate with each other during the journey, SDN nodes that are placed on fog nodes, main SDN control that is also placed on fog nodes, the cloud, which is for huge computation and storage, as shown in Figure 3. We have vehicles from $V_{1}$, $V_{2}$, $V_{3}$, ⋯, $V_{n}$; furthermore, we have SDN nodes from $SDN_{1}$, $SDN_{2}$, $SDN_{3}$, ⋯, $SDN_{m}$. We have Fog nodes from Fog${}_{1}$, Fog${}_{2}$, Fog${}_{3}$, ⋯, Fog${}_{k}$, and cloud nodes from $C_{1}$, $C_{2}$, $C_{3}$, ⋯, $C_{j}$. Vehicles send and receive messages such as $M_{1}$, $M_{2}$, $M_{3}$, $\cdots ,M_{s}$. The following Table 2 is used for the abbreviations used in the proposed model:

Our proposed model first gets messages from vehicles through nearby gateways, which are placed into fog nodes. Messages are categorized into safety and non-safety according to the nature of messages and deadline, and size. The concerned SDN controller performs necessary action and returns the result to vehicles otherwise sent to the cloud for storage and necessary action. The following Algorithm 1 is used for messages receiving and giving priority to them.
**Algorithm 1:** Vehicles Messages receiving and priority allocation based on message nature**Input:** (Vehicles Messages) **Output: **$W_{1}$ & $W_{2}$ (Messages Priorities)
  For **1** to **n**   $\Omega =M_{s}$ //Receive Messages from $V_{i}$ (*i* = 1 to n) and assign to Ω  End of for loop  For 1 to n Find Weight of Ω // according to message nature   **if (**Ω == **010 or** 011 **or** 012 **or 013 or** 014 **or** 015 **or** 016**)**   **then** assign **$W_{1}$** in ascending order    Else **$W_{2}$**   Return **$W_{1}$ & $W_{2}$**  End of for loop


The following Algorithm 2 is used to calculate messages priority based on deadline and size:
**Algorithm 2:** Vehicles Messages receiving and priority allocation based on deadline and size**Input:** (Vehicles Messages) **Output:**
$W_{1}$ & $W_{2}$ (Messages Priorities)
  For **1** to **n**   Ω = $M_{s}$//Receive Messages from $V_{i}$ (*i* = 1 to n) and assign to Ω  End of for loop  For 1 to n Find Weight of  Ω according to message nature   W = Deadline * Size  If W =< Deadline * Size of theTable 1**    then** assign $W_{1}$ in ascending order   Else $W_{2}$   Return $W_{1}$& $W_{2}$  End of for loop


   Algorithm 1 input consists of vehicle messages, and the output consists of $W_{1}$ & $W_{2}$ (weighted priority). Algorithm 1 consists of nine steps in which we used two linear for loops. The first for loop is used for messages/data receiving at SDN Controller, then the second for loop is used for priority calculation of the received messages according to the messages nature, as provided in Table 1. The algorithm at the end returns two lists of messages and further forward the messages to vehicles and to the cloud for storage, which is provided in Algorithm 3. Algorithm two is used for messages received at SDN nodes placed on fog nodes; the input consists of vehicle messages, the output consists of two lists. One is of priority basis messages. The other list consists of normal messages. Messages priority is calculated according to deadline and size; priority is calculated of the generated messages, if the priority is less or equal to the priority of deadline and size of the messages that are given in the table, then we assign it to list 1 otherwise to list 2.

The following Algorithm 3 is used for messages forwarding according to priority assign to them:
**Algorithm 3:** Messages forwarding to vehicles for information and cloud**Input:**$W_{1}$ & $W_{2}$ (Two list one of safety messages while the other one of non-safety messages) **Output:** Sending messages receipt and database storage
  For **1** to **n**   Send $W_{1}$ (**ascending order**)//Send priority basis/safety messages   Send to Fog Nodes by using Algorithm 4  End of for loop  For **1** to **n**   Send $W_{2}$ (**first come first serve basis**)   Send to Fog Nodes by using Algorithm 4  End of for loop  Save record to cloud for future use


   Algorithm three works based on received messages; the input consists of two lists from Algorithms 1 and 2. The first safety messages that are provided in Table 1 are sent. Then, the second list of normal messages is sent according to a first-come and first-serve basis.
**Algorithm 4:** Fault-Tolerance Mechanism for SDVN**Input:**$W_{1}$ & $W_{2}$ (Two list one of safety messages while the other one of non-safety messages) **Output:**
***£*** (Successful messages)
  For **1** to **n**   If Source received Ack   Then assign $M_{i}$ to ***£*** (Successful messages) // No action   Else   {   assign $M_{i}$ to ϕ (Failed tasks/messages) // resend messages   Call Algorithm 3   }  End of for loop  Return *£*


In step 3, we call Algorithm 4 for fault tolerance; if any message failed and did not reach the proper destination, then we provided a mechanism in algorithm four that will solve it accordingly.

Algorithm 4 is provided to tackle fault tolerance of the sent messages of vehicles. Algorithm 4 is used for failed and successful tasks; if the source receives the acknowledgment, then we assign it the list of successful messages; otherwise, we assign it to failed tasks/messages list and further resend according to Algorithm 3.

## 5. Simulation Setup and Results

This section consists of resource modeling, application modeling, performance parameters, and results. Results are provided based on different scenarios.

### 5.1. Resource Modeling

For the implementation and simulation of the proposed model, we need a real VANET environment, but a real environment is costly and cannot be created easily. So, for a simulation of the proposed model, we used CloudSim [32] & iFogSim [33]. Further pricing mechanism and resources allocation mechanism was obtained from [34,35,36]. We used vehicles for sending messages, SDN nodes, SDN controller, fog nodes in which we placed SDN nodes, and Cloud for heavy processing and storage. Further detail of the simulation environment is provided in Table 3.

### 5.2. Application Modeling

Multiple vehicles are considered to send messages to nearby fog nodes in which SDN nodes and controllers are fixed. The following Table 4 consists of the application model for the proposed model:

The following Table 5 consist of each datacenter power that will be used for tasks execution:

### 5.3. Simulation Process

In the simulation of the proposed model first, we created datacenters of clouds and their sub datacenters of fog. We created different vehicles which send data to nearby RSU/Gateways. Random tasks. Tasks are also created randomly as provided in the above table. Randomly the messages/data are sent to RSU/Gateways by the vehicles. The RSU/Gateway prioritizes the data in safety and non-safety messages by using a priority algorithm. The RSU/Gateway further forwards safety messages to the fog node while non-safety messages to the cloud. Fog node used the least priority first policy for the execution of the data while Cloud used FCFS scheduling model for execution of the offloaded data. From the different simulation/implementation runs, we obtain data and evaluate the data for further analysis, as provided in Section 5.5.

### 5.4. Performance Parameters

The following performance parameters are used to evaluate the proposed model:

#### 5.4.1. Response Time

Response time is the actual time of the resource’s response to the vehicles sent messages. Response time is calculated according to the following (1):

(1) $$ℜp=\mathrm{Communication}\;\mathrm{from}\;V{}_{i}\mathrm{to}\;\mathrm{Fog}\;\mathrm{Node}+\;\mathrm{Service}\;\mathrm{Time}+\mathrm{Communication}\;\mathrm{from}\;V_{i}\;\mathrm{to}\;\mathrm{Cloud}$$

#### 5.4.2. Execution Time

Execution time of the vehicles messages is the total time taken by the execution node to prioritize it and forward it to the destination.

(2) E=Service Start Time−Finish Service Time

#### 5.4.3. Tasks/Messages Failure Ratio

Messages failure ratio is the actual messages that failed to send and did not receive via the destination party. The following (3) is used to calculate the tasks/messages failure ratio.

(3) F=No of failed messages∗100/Total no of sent messages

### 5.5. Results Comparison

The following scenarios are created to evaluate the proposed model:

#### 5.5.1. Response Time Comparison with Random and Previous Work of Safety and Non-Safety Messages

**Figure 4:**
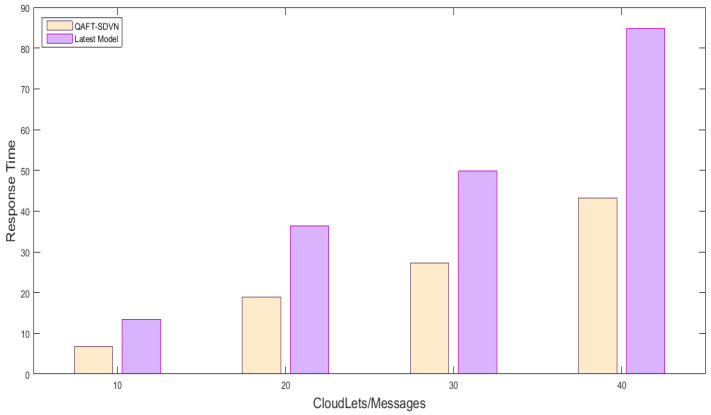
Response time comparison of proposed model with the latest models of safety messages.

**Figure 5:**
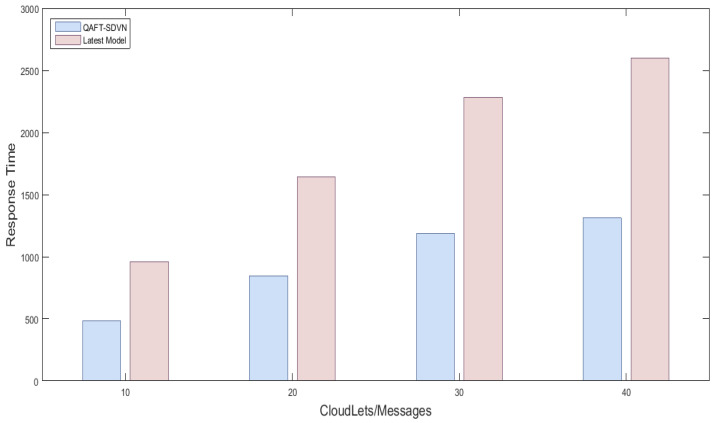
Response time comparison of proposed model with the latest models of non-safety messages.

We created one cloud node with a data center; the details are provided in Table 3 and Table 4. Three fog nodes are created with equal specification and placed three SDN nodes in which one was considered as SDN main controller. 15 vehicles randomly sent messages, and we observed their response time using fog nodes and fog nodes. Figure 4 shows the response time comparison of the proposed model with the latest work using fog and without fog nodes. Figure 4 x-axis shows No messages/Cloudlets/Tasks executed, and the y-axis shows response time milliseconds. We sent the first 10 messages to fog and then to cloud, then we sent 20 messages, 30 messages, and 40 messages. Due to fog usage, we reduced the response time of safety messages by up 50%, which is sufficient for vehicles in an emergency. So, the proposed QAFT-SDVN model perfumed very well in terms of response time while forwarding safety messages. The other non-safety messages were sent by FCPS methods whose response time was also reduced by fog nodes. Similarly, as shown in Figure 5, response time comparison non-safety messages are provided, and the results show that our proposed model performed well, as we used fog nodes for SDN technology instead of the cloud.

#### 5.5.2. Effect of the Execution Time of Latest Model with the Proposed QAFT-SDVN Model

**Figure 6:**
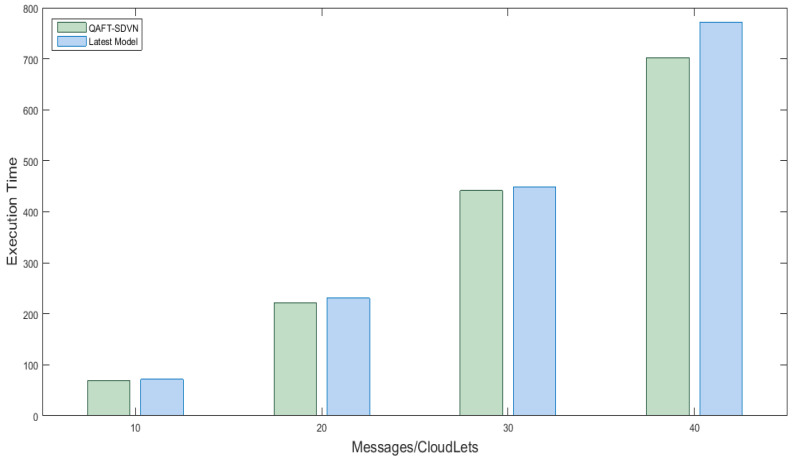
Execution time of safety messages in milli seconds.

In this scenario, fifteen vehicles were created, one cloud datacenter, three fog nodes placed with SDN nodes. Different messages are created and sent to fog nodes and then to the cloud, and their execution time is analyzed and compared with work already done in [27,28]. Figure 6 x-axis shows no messages from vehicles while the y-axis shows execution time taken by the messages. The execution time of the proposed model and the available work is the same, but our proposed model reduced execution time up to 4% by using efficient priority algorithms to categorize safety and non-safety messages.

#### 5.5.3. Tasks/Messages Failure Ration Comparison with Previous Work

**Figure 7:**
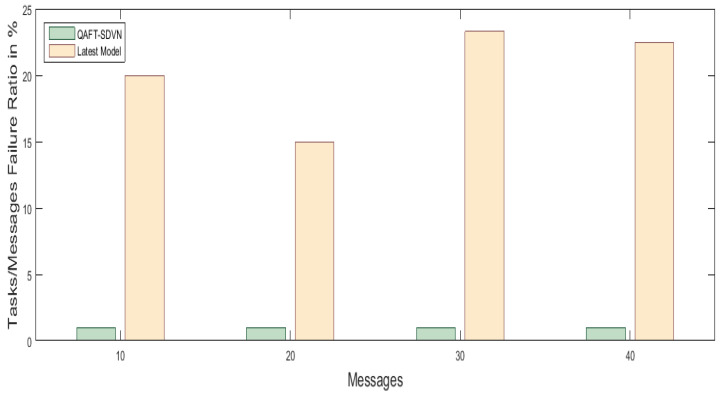
Tasks/Messages failure ratio comparison with available work.

In this scenario, we consider the same resources like the above scenarios. Here, we analyzed our Algorithms 3 and 4 for tasks/messages failure ration calculation. Our proposed model did not drop any message using a fault tolerance mechanism, while the previous work [27,28] randomly dropped 20%, 15%, 23.3%, and 22.5% messages, as shown in Figure 7.

### 5.6. Simulation Experiment/ Illustrative Example

In the experiment, we considered five vehicles with ten different messages, as shown in the following tables, as safety messages and non-safety messages are stored:

In the above two tables, safety and non-safety messages are stored and received from vehicles. In the simulation, we defined safety and non-safety messages with ID No to process further in Table 6 and Table 7. After receiving messages from vehicles, our Algorithm 1 checks the nature of the message and stores them in Table 6 and Table 7 accordingly. Algorithm 2 is used to prioritize the Algorithm 1 out tables and assign priorities as shown in Table 6 and Table 7 columns as “Priority”. Algorithm 3 sends Table 6 data to fog in ascending order as if any Vehicle message priority value is less than the message will be sent first. And Table 7 data were sent to the cloud for execution. After the assignment of the messages to their assigned execution machines the Algorithm 4 checks for fault tolerance. If any message fails to offload, then Algorithm 4 resends the failed message until successful offloading to the execution server. By priority basis scheduling and fault tolerance, the proposed model reduced energy consumption and messages failure ratio as compared with the latest work.

## 6. Discussion

In this section, we discussed the provided result in a detailed and comprehensive manner. The proposed model reduced response time by using fog in the middle of the RSUs and Cloud. As when data was sent to the cloud then it was taking so long due to the heterogeneous nature of the cloud. In the cloud basically, the services/servers are placed for way from the users/clients. So, as compared with QAFT-SDVN’s latest model, our proposed model reduced the 50% response time of the safety messages, while up to 20% of the non-safety messages due to load division on fog and cloud nodes. The result analysis shows that task failure was a drawback of the traditional models, and we reduced the tasks failure ratio up to 15% as compared with non-fault tolerant models. We have used the task retry mechanism and node retry mechanism when tasks are failed. Energy consumption is also reduced due to reducing the communication cost of the priority messages up to 25%.

## 7. Conclusions

In this paper, we provided QoS aware and fault tolerance-based software-defined vehicular networks using Cloud-fog computing (QAFT-SDVN). The proposed model communicates vehicle messages through SDN nodes which are placed on fog nodes. SDN controllers receive messages from nearby SDN units and prioritize the messages in two different ways. One is message nature-based, while the other one is based on the deadline and size of the messages. SDN controller categorized in safety and non-safety messages and forward to the destination. After sending messages to their destination, we check their acknowledgment; if the destination receives the messages, no action is taken. Otherwise, we use a fault tolerance mechanism, in which we re-transmit the messages. The proposed model is implemented, evaluated, and compared with the latest models. The results show that our proposed model decreased response time by 50% of the safety and non-safety messages by using Fog nodes for the SDN controller. Furthermore, we reduced the execution time of the safety and non-safety messages by up to 4%. Similarly, compared with the latest model such as the QAFT-SDVN model, we reduced the task failure ratio by 20%, 15%, 23.3%, and 22.5% by using the fault tolerant technique, Fog technologies, and messages nature-based priority. In the future, we will work on the vehicle’s mobility and connection with the different RSUs of vehicles. In the future, we will also need a security model for the proposed system. 

## Figures and Tables

**Table 1:** Literature review models with comparison.

Model Design	Model Name	Model Structure	Advantages	Disadvantages
VANET Models	VANET Based Mobile Ad-hoc Network	Vehicular ad-hoc systems for vehicles using wireless technology using mobile cellular system for communication	Efficient for movable vehicles for sharing important information	Dynamic topology updating and connection was a problem
	Safety and Non-Safety Messages VANET Architecture	Priority Basis vehicular ad-hoc network for the vehicle to vehicle (V2V) communication	An efficient model for important messages by using priorities	Difficult to categorize safety and non-safety messages
SDN Models for Vehicles Communication	Open Flow Model	Software Defined Networking Based V2V Model using wireless technology	Efficient for cross layer devices and compatible with different machines	Complex, difficult to implement in real world
	RSU Based Model	SDN Based Roadside Unit Vehicular Network Architecture	The communication can be delivered to multiple vehicles at a time	Difficult to find shortest RSU for multiple vehicles
	SDNE Model	Services provision to vehicles to their nearby edge architecture	Reduced response time and energy consumption	Node energy is the problem of edge services
	RTISAR Algorithm	QoS aware model for V2V communication	Reduced connectivity and response time	Point to Point link is a drawback of the system
SDVN Models for Vehicles Communication	Topology based SDVN Model	SDN and VANET based model using dynamic path selection of vehicles	Reduced communication cost using unicast and multi-cast models	Difficult to combine SDV with VANET
	Controller based SDVN Model	Dynamic Controller based V2V model	Efficient for roadside traffic	Controller location placement is dependent on the performance of the model
	Multi-Access Edge Model	Put services on different edges of the roadside vehicles	Efficient for important messages as the network has low latency	Putting edge devices on different locations is costly
	SDN Environment Based SDVN Model	Open Flow Switches based SDVN model	Accommodate more vehicles at a time using switches	Complex architecture and experience more delay
SDVN Scheduling Models	UVN Based Model	Unmanned Ariel Network, architecture less model, using zone-based data offloading model	Efficient for emergency zone data	Other than emergency zone data will have to experience unlimited delay
	Priority Basis RSA Algorithm	FCFS, SJF based model for urgent, least urgent data	Reduced energy consumption	Priority assigning is difficult
	D*S Algorithm	Priority based model based on deadline and size	Reduced communication cost of urgent messages	Increased communication cost of normal messages
	Collective Scheduling Algorithm	Used different scheduling models for priority-based messages	Efficient for important and urgent messages	Slow for normal messages

**Table 2:** List of abbreviation used in the proposed model.

Abbreviation	Standsfor
*£*	Successfulmessages
φ	Failedtasks/messages
$M_{i}$	Messagesofvehicles
Ack	Acknowledgment
SDVN	SoftwareDefinedVehicularNetwork
$W_{1}$ & $W_{2}$	Safetyandnon-safetymessages
Ω	Receivedmessagesforforwarding
SDN	SoftwareDefinedNetworking
*M*	Message
*C*	CloudComputing
Fog	FogComputing
${ℜ}_{p}$	Responsetime/ExecutionTime
QoS	QualityofService
VANET	VehicularAd-HocNetwork
*F*	TasksFailureRatio

**Table 3:** Message types with deadline and size.

S. No.	Message with ID	Deadline (Seconds)	Size (Bits)
1	Rescue call 010	67	2300
2	Hospital Emergency call (011)	65	2100
3	Call to nearest traffic signal (012)	77	2800
4	Police help for accident (013)	61	2000
5	Nearest petrol help (014)	72	2700
6	Robbery (015)	59	1900
7	Murder information (016)	60	2100

**Table 4:** Application modeling.

Setup	Power	Tasks/Messages	Users/Broker	VMs
Cloud Setup	One datacenter	40 Cloudlets	One Broker	One VM
Fog Setup	Three fog nodes	–	–	3 SDN
Vehicles Setup	15 Vehicles	40 messages	15 brokers	–

**Table 5:** Datacenter detail.

S. No.	Configuration	Detail
1	DC Architecture	x86
2	DCRAM (MB)	512
3	DC Storage (MB)	2048
4	DC OS Hypervisor	Xen
5	DC Computation Power (MIPS)	1000/sec
6	DC Bandwidth (MBPS)	1000

**Table 6:** Safety messages.

S. No.	Vehicle No.	Message	Deadline	Size	Deadline & Size	Priority No.
1	V1	Robbery (015)	59	1900	112,100	P1
2	V2	Hospital Emergency call (011)	65	2100	136,500	P4
3	V3	Police help for accident (013)	61	2000	122,000	P2
4	V4	Murder information (016)	60	2100	126,000	P3
5	V5	Rescue call 010	67	2300	154,100	P5

**Table 7:** Non-Safety Messages.

S. No.	Vehicle No.	Message	Deadline	Size	Deadline & Size	Priority
1	V1	Call to nearest traffic signal (012)	77	2800	215,600	P4
2	V2	Nearest petrol help (014)	72	2700	194,400	P1
3	V3	Call to nearest traffic signal (012)	75	2800	210,000	P3
4	V4	Nearest petrol help (014)	74	2700	199,800	P2
5	V5	Call to nearest traffic signal (012)	78	2800	218,400	P5
